# Application of the regression of offspring on mid-parent method to detect associations between single-nucleotide polymorphisms and the beta 2 electroencephalogram phenotype in the COGA data

**DOI:** 10.1186/1471-2156-6-S1-S56

**Published:** 2005-12-30

**Authors:** Marie-Hélène Roy-Gagnon, Rasika A Mathias, Alexander F Wilson

**Affiliations:** 1 Genometrics Section, Inherited Disease Research Branch, National Human Genome Research Institute, National Institutes of Health, 333 Cassell Dr., Suite 2000, Baltimore, Maryland 21224, USA

## Abstract

The beta 2 electroencephalogram (EEG) phenotype is used as a quantitative measure related to alcoholism, and evidence of linkage and association has previously been reported in the Collaborative Study on the Genetics of Alcoholism data. In this study, associations between the beta 2 EEG phenotype and single nucleotide polymorphisms from whole-genome Illumina and Affymetrix panels were investigated with the regression of offspring on mid-parent method to identify significant genetic effects and to estimate their heritability. Separate regressions on father and mother were performed to identify parent-specific effects. Estimates of the heritability of the beta 2 EEG phenotype were 0.68 ± 0.12 and 0.52 ± 0.07 based on father-offspring and mother-offspring pairs, respectively. Significant associations at the 0.0005 level, some of which were parent-specific, were found on chromosomes 1, 2, 5, 6, 7, 8, 11, 12, 15, 16, 17, 18, and 19 with heritability attributable to each SNP ranging from 0.01 to 8%.

## Background

The first component of a trilinear singular value decomposition of the beta 2 band electroencephalogram (EEG) waves has been used as an alcoholism-related quantitative trait. Linkage and association were reported between this phenotype and markers in the chromosome 4p12 GABA_A _receptor gene cluster in data from the Collaborative Study on the Genetics of Alcoholism (COGA) [1-3]. Song et al. [4] reported parent-of-origin effects for the GABA_A _cluster on chromosome 15q11.2-q12 using a qualitative alcoholism phenotype in these same data.

The goals of this study were: 1) to use the regression of offspring on mid-parent (ROMP) [5,6] to test for associations between the beta 2 EEG phenotype (ECB21) and the single-nucleotide polymorphism (SNP) based genome screens from Illumina and Affymetrix in the COGA data; and 2) to investigate parent-specific effects that may suggest a genetic parent-of origin effects (transmission from specific parent), parental imprinting or environmental effects (such as intrauterine effects).

## Methods

Departure from Hardy-Weinberg equilibrium was tested in the founders for each SNP using a chi-square test. The average ± standard deviation minor allele frequency in the founders was 0.39 ± 0.09 (range: 0.08–0.5) for the 4,596 Illumina SNPs and 0.27 ± 0.14 (range: 0–0.5) for the 10,810 Affymetrix SNPs. The ROMP method was used to estimate the heritability of the ECB21 phenotype and to test for association with each SNP from the two panels.

ROMP is an extension of the traditional regression of offspring on mid-parent that provides, in addition to the estimate of the heritability (*h*^2^) of a quantitative trait, a test of association between the trait and a locus, along with an estimate of the locus-specific heritability (*h*_L_^2^). The association test is obtained by adding a locus effect (the genotype of the offspring at the locus) in the regression model. The change in the regression coefficient of the mid-parent value when the locus is added to the model is the locus-specific heritability estimate. ROMP is performed in parent-offspring trios and requires phenotype data on the parents and their offspring but genotype data on the offspring only. The X-chromosome markers were not included in these analyses.

ECB21 was first adjusted for age at exam (ERP age), ERP age squared, and sex. This was done in a separate multiple linear regression model including all offspring. The residuals from this regression were then used in the ROMP analysis. The genotype of the offspring at each SNP was entered in the ROMP analysis assuming an additive allelic effect. ROMP was performed using all offspring from each sibship and a randomly selected single offspring to ensure independent parent-offspring trios. A robust estimate of variance that takes into account the correlation between multiple sibs from nuclear families is not available yet for the locus-specific heritability.

SNPs with a minor allele frequency smaller than 0.1 and SNPs for which Hardy-Weinberg equilibrium was rejected at the 0.001 significance level were excluded from the analysis. Results from the association tests were considered significant if: 1) the *p*-value from the all-sib analysis was lower than 0.0005, and 2) the one-sib analysis also showed a trend toward significance (*p*-value < 0.1).

Separate regressions of offspring on mother (ROOM) and on father (ROOF) were also performed to investigate parent-specific effects. In these regressions, twice the regression coefficient is the heritability estimate. All-sib and one-sib analyses were performed in the same manner as for ROMP, and the same criteria were applied to determine significance. Permutation tests were used to assess the significance of these parent-specific effects. The father-offspring pairs were combined with the mother-offspring pairs, duplicating the offspring's information if the phenotype was available on both parents. A variable indicating the parent's sex was created. *p*-Values were obtained from 10,000 permutations of the parent's sex, keeping the other variables fixed, by counting the number of permuted samples for which the father-mother difference in heritability estimates divided by their respective standard errors exceeded the difference observed in the original sample.

SAS Release 8.2 [7] was used for data management, descriptive statistics, and Hardy-Weinberg tests. ROMP was implemented in R [8].

## Results

Analyses were restricted to the 1,074 non-Hispanic White subjects (67% of the total sample). These subjects belonged to 119 families ranging in size from 4 to 32 subjects for a total of 270 sibships with an average of 3 offspring (range: 2–10). Of the 13 quantitative traits released in the COGA data, we focused on ECB21, for which linkage and association were previously reported. All SNPs in the Affymetrix and Illumina panels were tested individually. Affymetrix and Illumina SNPs were then combined using the physical map (Build 34 of dbSNP [9]) to facilitate one single graphical overview of all results. Map positions for 392 of the Affymetrix SNPs could not be found and these SNPs were not included in the results.

Among the 1,074 (112 probands and 962 family members) non-Hispanic White subjects of COGA, 79% of the probands and 44% of the family members were male. The average ERP age ± standard deviation was 42.5 ± 12.8 and 32.2 ± 8.0 for male and female probands, respectively, and 37.5 ± 14.6 and 39.7 ± 14.5 for male and female family members, respectively. The average ECB21 was 13.8 ± 4.9 and 13.6 ± 4.9 for male and female probands, respectively, and 13.9 ± 4.8 and 16.4 ± 5.9 for male and female family members, respectively.

In a multiple linear regression model, sex, ERP age, and ERP age squared were strongly associated to ECB21. Consequently, ECB21 was adjusted for these variables. The heritability of age- and gender-adjusted ECB21 was high (0.450 ± 0.066) and statistically significant (*p*-value < 0.001). A total of 236 trios were used in the ROMP analysis. There were many instances with only one phenotyped parent, resulting in 295 father-offspring pairs and 359 mother-offspring pairs for the ROOF and ROOM analyses, respectively. The trait heritability estimate for father-offspring pairs was slightly higher (0.675 ± 0.123) than for mother-offspring pairs (0.518 ± 0.072), but the confidence intervals overlapped (Permutation test *p*-value = 0.2908).

**Figure 1:**
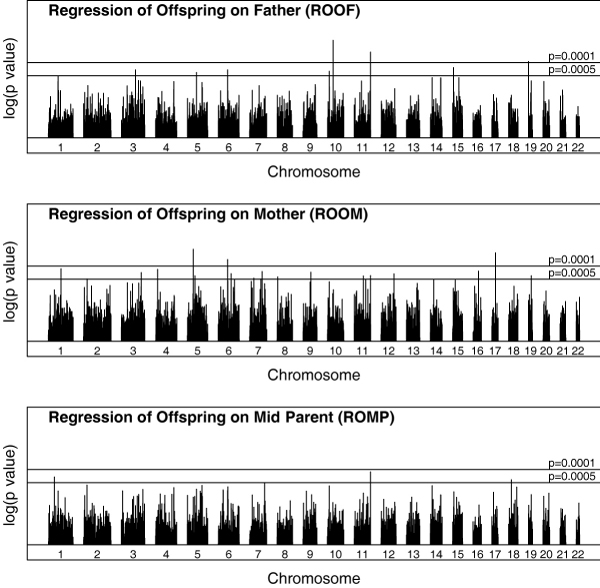
Results of tests of association with ECB21 for all SNPs in the non-Hispanic White subjects.

Figure 1 presents the results of the association analysis using ROOF, ROOM, and ROMP approaches, and using all sibs from each nuclear family. A total of 33 of these tests were significant at the 0.0005 level with either ROOF, ROOM, or ROMP. Twenty-four of the 33 also had a corresponding one-sib analysis *p*-value smaller than 0.1. These 24 results (23 SNPs) were taken to be significant (Table 1). A total of 39,342 tests were performed, yielding an observed proportion of significant results of 0.0006 with the significance criteria described above. The proportion of significant tests was 0.0157, 0.0056, and 0.0012 when the threshold for the all-sib analysis was changed to 0.05, 0.01, and 0.001, respectively.

There were multiple significant SNPs on chromosomes 1, 5, 6, 7, 11, and 19, although these were physically too far apart to be considered single loci except for a 103-kb region on chromosome 5 (SNPs tsc1780606, tsc0644829, rs1020661). Among the significant results, the proportion of the total heritability attributable to the locus ranged from 0.01% to 8%, with 17 and 3 SNPs having locus-specific heritability greater than 1% and 5%, respectively.

Twelve SNPs had similar trends (*p*-value < 0.05 was considered a trend in the parent-specific analysis) in both father- and mother-offspring analyses. Mother-offspring associations alone were observed for the remaining 11 SNPs. However, permutation tests indicated that the father-mother difference in locus-specific heritability estimates was significant at a 0.001 level for only one of these 11 SNPs (tsc0972067).

## Conclusion

Using ROMP, significant associations at the 0.0005 level were detected between the beta 2 EEG phenotype and 23 SNPs on chromosomes 1, 2, 5, 6, 7, 8, 11, 12, 15, 16, 17, 18, and 19. None of these SNPs are located in the areas where evidence for linkage and/or association was previously found in the COGA data. Four SNPs in the panels are located in the GABA_A _receptor gene cluster within the GABRA4 and GABRB1 genes. Associations were previously reported with the GABRB1 microsatellite marker and with SNPs in the GABRA2 gene of that cluster. These 4 SNPs were also analyzed using ASSOC (S.A.G.E. 4.3 [10]) and no significant association was found (*p*-value > 0.1). The SNPs for which significant associations were found in this study are not located within or near any of the known candidate genes for alcoholism [11,12]. Overall, the results from these analyses do not corroborate the previously reported linkage and association results.

The estimate of locus-specific heritability may provide insight on the importance of each SNP. Three out of the 23 SNPs that showed significant associations had a locus-specific heritability estimate between 5 and 8%. The separate regressions of offspring on father and mother were performed as a screening tool in an attempt to detect any difference in parental effect. One of the 23 SNPs, tsc0972067, showed a significant association only in mother-offspring trios, suggesting some type of maternal effect.

## Abbreviations

COGA: Collaborative Study on the Genetics of Alcoholism

EEG: Electroencephalogram

ROMP: Regression of offspring on mid-parent

ROOF: Regressions of offspring on father

ROOM: Regressions of offspring on mother

SNP: Single-nucleotide polymorphism

## Authors' contributions

All authors were involved in the conception of the study. M-HR-G and RAM analyzed the data and wrote the draft manuscript. All authors provided comments on the draft manuscript, and read and approved the final manuscript.

## Figures and Tables

**Table 1:** Summary of significant tests of association for ECB21 in non-Hispanic White subjects

SNP	Chr.	Location (Mb)	ROOF	ROOM	ROMP	*p*-Value of a permutation test for a parent-specific effect
				
			*h*_L_^2 ^± SE	*h*_L_^2 ^± SE	*h*_L_^2 ^± SE	
tsc0499325	1	63.98	-0.023 ± 0.007	0.025 ± 0.008 *	0.017 ± 0.005 ***	0.0399
rs952499	1	94.03	0.001 ± 0.0003 ***	0.024 ± 0.011	0.018 ± 0.006 *	0.3836
tsc0523529	2	26.24	0.007 ± 0.003	0.047 ± 0.013 ***	0.002 ± 0.001	0.2557
tsc1780606	5	57.09	-0.014 ± 0.006	0.039 ± 0.010 ****	-0.001 ± 0.0002	0.0230
tsc0644829	5	57.14	-0.032 ± 0.012	0.017 ± 0.005 ***	-0.010 ± 0.004	0.1764
rs1020661	5	57.20	-0.001 ± 0.001	0.035 ± 0.008 ****	0.006 ± 0.002	0.0283
rs164561	5	68.36	0.014 ± 0.009	0.015 ± 0.004 ***	0.010 ± 0.004 *	0.2461
rs1445748	5	83.66	0.042 ± 0.012 ***	0.020 ± 0.007	0.020 ± 0.006 *	0.6491
tsc0044751	6	83.73	0.051 ± 0.014	0.057 ± 0.014 ****	0.054 ± 0.017 *	0.7666
rs1033391	6	113.81	0.039 ± 0.017	0.0001 ± <0.0001 ***	0.020 ± 0.007 *	0.5177
tsc0066015	7	34.39	0.027 ± 0.020	0.0005 ± 0.0001 ***	0.009 ± 0.003	0.2455
tsc0065157	7	106.76	0.040 ± 0.020	0.013 ± 0.004 ***	0.016 ± 0.009	0.3011
rs868053	7	120.33	0.076 ± 0.043	0.024 ± 0.006 ***	0.043 ± 0.020	0.1620
rs899145	8	4.57	0.030 ± 0.030	0.015 ± 0.004 ***	0.020 ± 0.009	0.0693
rs561646	11	85.47	0.047 ± 0.018	0.006 ± 0.002 ***	0.022 ± 0.009	0.5141
rs2076848	11	134.21	0.019 ± 0.005	0.007 ± 0.002 ***	0.005 ± 0.001 ***	0.8046
tsc0972067	12	112.64	-0.018 ± 0.006	0.079 ± 0.021 ***	0.031 ± 0.013	0.0006
rs278357	15	29.21	0.049 ± 0.013 ***	0.017 ± 0.008	0.012 ± 0.005	0.3159
tsc1645889	16	62.27	0.014 ± 0.007	0.032 ± 0.009 ***	0.015 ± 0.007	0.1901
tsc0016820	17	43.47	0.002 ± 0.001	0.035 ± 0.008 ****	0.010 ± 0.004	0.1005
tsc0009027	18	23.64	0.008 ± 0.003 *	-0.013 ± 0.005	0.007 ± 0.002 ***	0.2327
rs1020382	19	0.22	0.053 ± 0.013 ****	0.031 ± 0.011 *	0.049 ± 0.021	0.3702
rs7937	19	45.99	0.071 ± 0.021 **	0.009 ± 0.003 ***	0.036 ± 0.012 *	0.8539

*p*-Values from all-sib analyses: * < 0.01; ** < 0.001; *** < 0.0005; **** < 0.0001. Only the results for which the corresponding one-sib analysis *p*-value (not shown) was < 0.1 were considered significant at any level and are marked with the star symbols.
